# Mutiple Ionization of Rare Gases by Electron Impact

**DOI:** 10.6028/jres.063A.015

**Published:** 1959-12-01

**Authors:** M. Krauss, R. M. Reese, V. H. Dibeler

## Abstract

Electron impact studies of multiple ionization processes in helium, neon, argon, and xenon appear to support theoretical conclusions that the threshold probability for *n*-fold ionization is proportional to the *n*th power of the electron energy in excess of the threshold energy. The probability law applies, for the cases studied, over a considerable energy range that, for all but He^2+^, includes the possible onset of more than one mode of ionization. The presence of a Boltzmann spread in the energy of the electron beam or specific focusing effects due to ion source geometry are found to affect only the foot of the probability curve. By the use of certain assumptions, an estimate is also made of the departure from a ^3^P ionization probability curve resulting from onset of ionization to the ^1^D and ^1^S states.

## 1. Introduction

Wannier [1]^1^ and more recently Geltman [2] have shown theoretically that the threshold probability for *n*-fold ionization by electron impact is proportional to the *n*th power of the electron energy in excess of the threshold energy. Fox has reported [3] experimental results for the retarding potential difference method which indicate that for some atoms, e.g., xenon, linear probability laws apparently hold even for multiple ionization.

The purpose of this paper is to report the results of a study of multiple ionization in several of the rare gases, helium, neon, argon, and xenon, in which the data seem to support the theoretical conclusions. Although a monoenergetic electron beam is not used in this work, an attempt has been made to establish the extent of the effect on the ionization probability curve resulting from the presence of a Boltzmann distribution in the electron beam and the possible onset of ionization to energy states above the ground state of the ion.

## 2. Experimental

Measurements were made by means of a conventional Nier-type mass spectrometer with a 6-in. radius of curvature previously described in some detail [4]. However, in the present work the ion detecting system was replaced by a 14-stage electron multiplier with accessory equipment for ion counting. Usually, the minimum statistically acceptable signal was 4 counts in 10 sec. Assuming a one-to-one correspondence in the multiplier, this is equivalent to about 6.5×10^−20^ amp. However, a comparison measurement at higher currents indicated an approximate ratio of 0.7 for output pulse per input pulse. Thus the threshold current is estimated to be about 10^−19^ amp. The resolving power was sufficient for good separation of the ^4^He^2+^ and ${\;}^{1}H_{2}^{+}$ ions.

Some measurements were made using a second sectorfield instrument with a 12-in. radius of curvature and an ion source differing in detail from that used above. The resolving power was sufficient to successfully separate the ^20^Ne^+^ ions near the threshold of the ^40^A^2+^ ion resulting from a trace of neon impurity in the argon.

All gases were the purest commercially available “Research Grade” materials stated by the supplier to contain less than 0.01 percent non-rare gas impurities.

## 3. Results and Discussion

**Figure 1:**
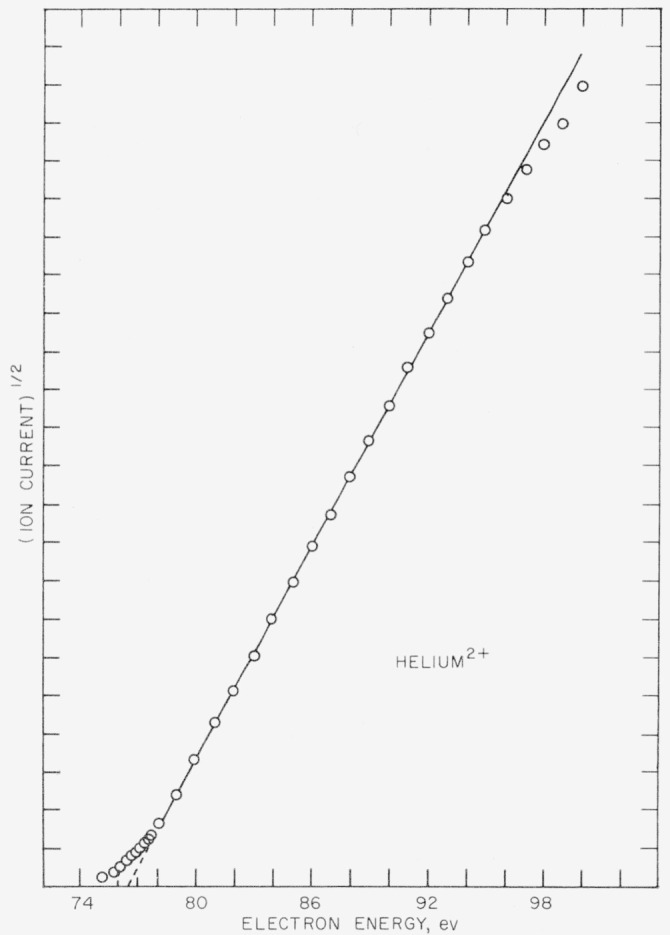
Ionization probability curve for the *He*^2+^ ion. The electron energy scale is uncorrected.

In figure 1, the square root of the observed ion current for the ^4^He^2+^ ion is plotted against the uncorrected electron energy. After an initial region of about 2ev above threshold, the curve is essentially linear for 18ev. This is in general agreement with the results for ^3^He^2+^ recently reported by Fox [3] in which the ionization probability curve follows a square law.

**Figure 2:**
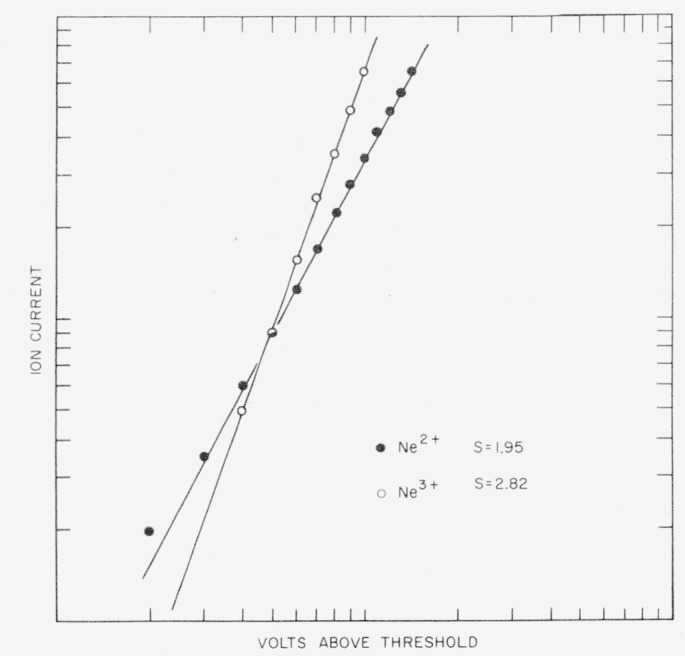
Log-log plots of the ionization probability curves of the *Ne*^2+^ and *Ne*^3+^ ions. The ordinate is different for each ion. Electron energy in excess of the critical voltage is plotted as the abscissa.

Figure 2 shows a log-log plot of the Ne^2+^ and Ne^3+^ ions. The ion current is different for the two curves. The observed slopes of 1.95 for the Ne^2+^ ion and of 2.82 for the Ne^3+^ ion indicate that essentially square and cube laws are involved in the ionization probability for doubly and triply charged ions. Similar results were obtained for argon. The slopes of approximately 2, 3, and 4 were observed for the A^2+^, A^3+^, and A^4+^ ions, respectively.

**Figure 3:**
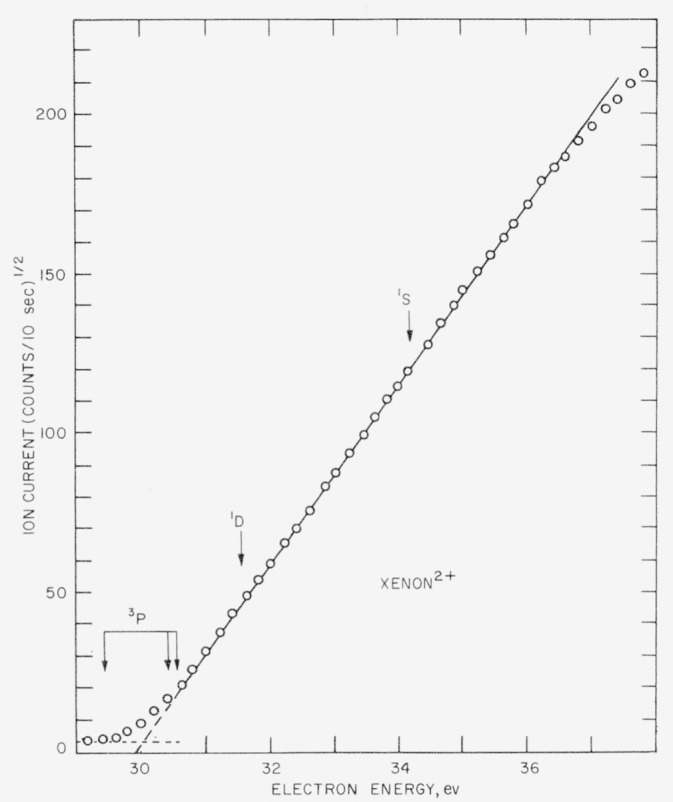
Ionization probability curve for the *Xe*^2+^ ion, showing the relative positions of the ^3^*P*, ^1^*D*, and ^1^*S* states. The electron energy scale is uncorrected.

The ionization probability curve for Xe^2+^ is given in figure 3 with the square root of the ion current again plotted against the uncorrected electron energy. The relative positions of the ^3^P, ^1^D, and ^1^S states are shown by arrows. After a short exponential foot, the curve is linear for nearly 7ev. Although any indication of onset of the ^3^P states above threshold may be concealed in the foot, there is no evidence for appearance of the ^1^D and ^1^S states. The apparent absence of onset of ionization to these states together with the indication of a square-law for the ionization probability function is at variance with the results reported by Fox [3].

The present results as well as those reported by Fox and coworkers indicate that if the probability of ionization can be considered a function of the series

$$\sum_{i=0}C_{i}{\left(V-V_{c}\right)}^{i+n}$$ (1)

then the first term dominates for a considerable voltage range. *V_c_* is the threshold value and *n* is found in this work to be equal to the degree of ionization and is generally found by Fox [3] to be one except for He^2+^. In the case of the rare gases this range is evidently several volts; and, as a consequence, we can employ the relations developed by Honig [5] to demonstrate the effect on the ionization probability curve of the Boltzmann spread in the electron beam.

$$\begin{array}{l} N(V)=C_{2}T^{2}\;\exp\;\left(\frac{-W-\eta }{kT}\right)2kT\\ \;\times [(V_{c}-V)+3kT]\;\exp\;\left(\frac{-V_{c}-V}{kT}\right)\\ \;\text{for}\;(V\leq V_{c}) \end{array}$$

and

$$\begin{array}{l} N(V)=C_{2}T^{2}\;\exp\;\left(\frac{-W-\eta }{kT}\right)\\ \;\times [6k^{2}T^{2}+4kT(V-V_{c})+{\left(V-V_{c}\right)}^{2}].\\ \;\text{for}:\;(V\geq V_{c}) \end{array}$$

**Figure 4:**
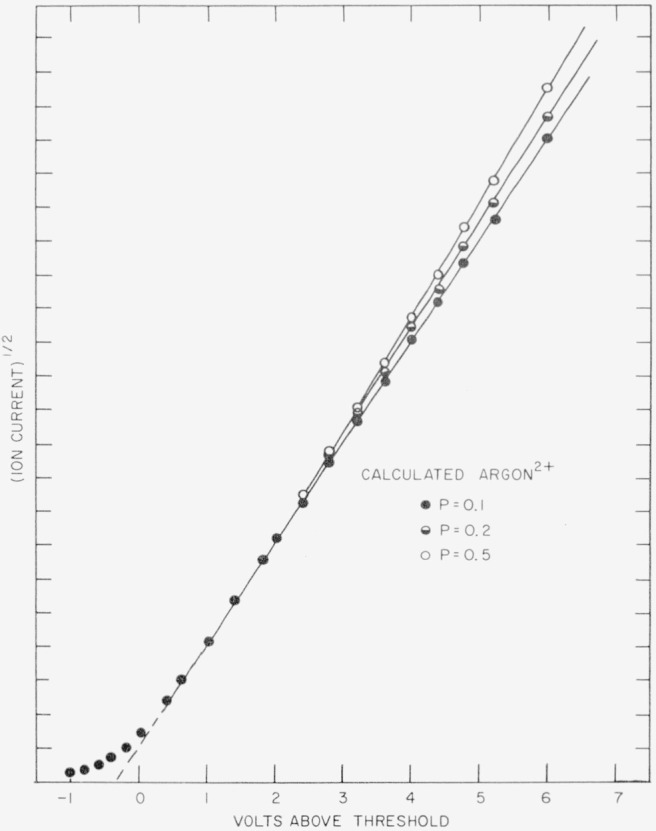
The calculated ionization probability curve for the *A*^2+^ ion, using Honig’s equations. The effect of ionization to ^1^D and ^1^S states is shown for several probability values.

This is illustrated in figure 4. The solid circles describe the calculated curve for doubly charged argon in which the square root of the ion current is plotted against the electron energy above the threshold or critical voltage. The characteristic foot and linear region indicate that the Boltzmann spread has little or no effect on the curve above the critical voltage (scale zero of the abscissa in figure 4.) Furthermore, if Honig’s equations are extended to include the voltage drop across the filament, the effect is merely that of reducing the critical slope with small change in the overall shape of the probability curve.

Other factors that may affect the shape of the ionization probability curve are the geometry and the focusing properties of the ion source. However, very nearly identical curves were obtained for Ne^2+^ and A^2+^ using two mass spectrometers with ion sources differing in geometric and in electron and ion focusing properties. Effects dependent upon such parameters are apparently confined to the foot of the curve.

Table 1 summarizes the several energy levels near the ground state for the doubly charged ions of Ne, A, Kr, and Xe. It is apparent that although figures 2 and 3 cover voltage intervals that include more than one possible onset, no indication of the excited states is observed. This may result from a very low ionization probability of the upper states compared with that of the ground state of the ion. Figure 4 illustrates the extent of the effect on the calculated ionization probability curve for the A^2+^ ion resulting from onset of the ^1^D and ^1^S states when the probability of onset of each magnetic sublevel of the ^1^D and ^1^S states is 0.1, 0.2, and 0.5 relative to that of the ^3^P_0_ state.

The experimental evidence is that it is only necessary that the proportionality constant for ^1^D and ^1^S be less than ½ of that of the ^3^P for onset of these states to be overlooked on a square root plot. The log-log plot is even less sensitive.

**Figure 5:**
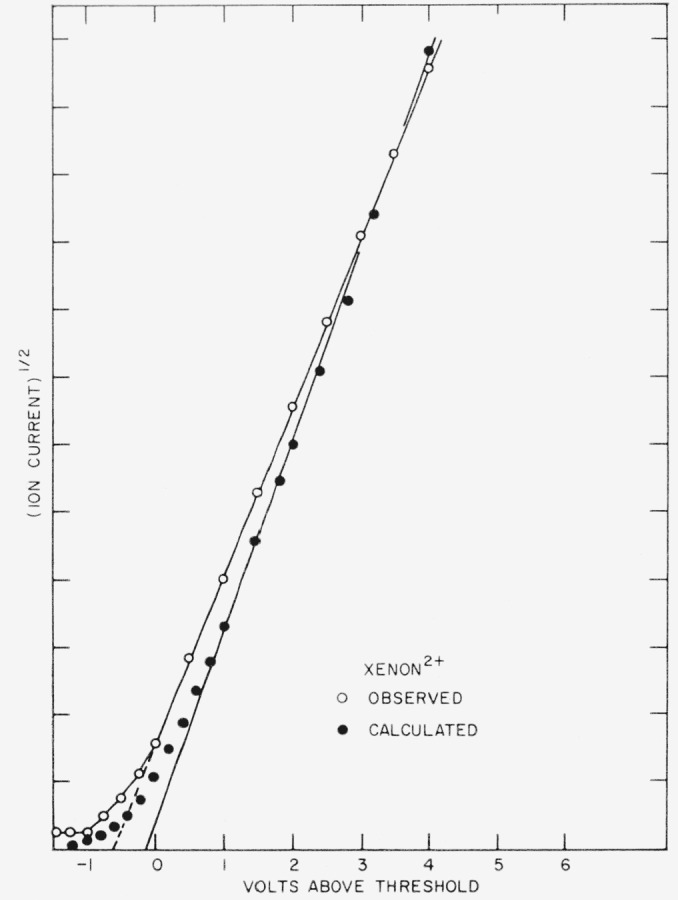
Calculated and observed ionization probability curves for the *Xe*^2+^ ion. The calculated curve is normalized to approximately the same slope to indicate the relative intervals of the exponential foot.

We also observe that the extent of the foot on a square root plot of the doubly charged ions is remarkably similar for all of the rare gases. The somewhat larger foot for He^2+^ may result from possible incomplete resolution of the H_2_^+^ ion impurity. The similarity is surprising in view of the very different multiplet splitting (see table 1). If the radial portions of the wave functions are the same for all *J* levels then the relative ionization cross sections to these levels are proportional to their weights; i.e., (2*J*+1). Calculated curves do show a larger foot for Xe^2+^ than for A^2+^ ions and, as figure 5 indicates, a larger calculated foot for Xe^2+^ than that observed experimentally. The experimental data of figure 5 is a replot of figure 3 with the calculated curve normalized to approximate the same slope.

The neon and argon multiplet levels are both well within the foot of the curve and in the present experiments would lead to identical curves in that region as observed. However, as table 1 indicates, the *J*=2 and *J*=0 ^3^P levels of xenon show a strong perturbation resulting from spin-orbital coupling. If, as indicated above, the excited states have smaller cross sections than the ^3^P state, the admixture of these configurations to the ground state wave function (due to the perturbation) would tend to reduce the *J*=2 transition probability. It is only conjecture, however, as to whether the reduction would be sufficient for the *J*=1 transition to dominate, but for the transition to the *J*=2 level to remain strong enough to permit the observation of the correct ionization potential. Further investigation of this point is required.

Some of the ambiguity of these measurements is avoided by selecting an atom the multiply-charged ions of which do not have excited states within a few volts of threshold. Sodium is an example and preliminary measurements [6] of this element and others of the alkali metal series indicate support for the observations reported here. A detailed report of further studies of the alkali elements will be submitted for publication at a later date.

## Figures and Tables

**Table 1:** Summary of several lowest energy levels for the doubly charged ions of the rare gases

State	*J*	Energy levels (cm^−1^)			
		
		Ne	A	Kr	Xe
^3^P	$\left\{ \begin{array}{l} 2\\ 1\\ 0 \end{array}\right.$	0	0	0	0
		650	1112.4	4548	9795
		927	1570.2	5313	8131
^1^D	2	25841	14010	14644	17100
^1^S	0	55747	33267	33079	37398

## Footnotes

1 Figures in brackets indicate the literature references at the end of this paper.
